# Clinical features and surgical outcomes in young children with focal cortical dysplasia type II

**DOI:** 10.1111/cns.13205

**Published:** 2019-08-01

**Authors:** Tian‐Shuang Wang, Qing‐Zhu Liu, Ming Liu, Qian Zhang, Ruo‐Fan Wang, Chong‐Wei Wu, Jie Zhang, Wen Wang, Tao‐Yun Ji, Xiao‐Yan Liu, Shuang Wang, Li‐Xin Cai, Yu‐Wu Jiang, Ye Wu

**Affiliations:** ^1^ Department of Pediatrics Peking University First Hospital Beijing China; ^2^ Children Epilepsy Center Peking University First Hospital Beijing China

**Keywords:** epileptic encephalopathy, focal cortical dysplasia type II, neurodevelopment, surgical outcomes, young children

## Abstract

**Aims:**

To investigate clinical characteristics and surgery outcomes of young children with focal cortical dysplasia (FCD) type II.

**Methods:**

Young children (onset age ≤6 years) with FCDII who underwent epileptic surgery in Children Epilepsy Center of Peking University First Hospital in 2014‐2018 were followed up for at least 6 months after surgery.

**Results:**

One hundred and twelve children with FCDII were included, with median age of onset 0.9 years (0.01‐5.9), who underwent surgery at 4.1 years old (0.8‐16.2). Focal seizures were most frequent (90.2%) and epileptic spasms presented in 23 (20.5%) cases. Epileptic encephalopathy was not uncommon (12.5%), associated with earlier epilepsy onset and higher rate of bilateral onset on ictal EEG (OR = 0.213, 9.059; *P* = .041, .004). At the last follow‐up, 88.4% achieved seizure‐free. Before surgery, 49.1% showed moderate/severe developmental delay, associated with earlier seizure onset and higher rate of history of epileptic encephalopathy (OR = 0.740, 5.160, *P* = .023, .042). For 48 children with preoperatively moderate/severe developmental delay, DQ rank at 6 months postsurgery was improved in only four cases.

**Conclusion:**

For young children with FCDII, they tend to present with epileptic encephalopathies and show moderate/severe developmental delay before surgery. The seizure outcome was favorable after surgery. For children with preoperatively moderate/severe developmental delay, developmental outcome at 6 months after surgery was not satisfactory.

## 1 INTRODUCTION

Focal cortical dysplasia (FCD) refers to a common spectrum of malformation of cortical development (MCD).^1^ It is the most common histopathologic finding in brain tissue obtained from epilepsy surgeries in children.^2^ Among 464 children who underwent epilepsy surgery in Children Epilepsy Center of Peking University First Hospital from May 2014 to April 2018, two hundred and seventeen (46.8%, 217/464) were confirmed to be FCD. FCD type II, which is characterized by a combination of dysmorphic neurons and balloon cells, is the most common type of FCD, accounting for 60.8% (132/217) of FCD cases. The age at seizure onset in patients with FCD II was reported to range from neonate to 53 years old.^3^, ^4^ For children with early‐onset epilepsy, longer exposure to seizures and epileptic discharges, FCD lesions, and multiple antiepileptic drugs might have detrimental effects on the developing brain. Especially, the epileptic activities may have adverse effects on cognitive and behavioral development beyond the FCD lesions and the condition can get worse over time, which also referred to as epileptic encephalopathies. And this phenomenon is more severe and common in infancy and early childhood.^5^ From the perspective of epileptic syndromes, some epileptic syndrome in infancy and childhood are classified as epileptic encephalopathy, such as infantile spasm (IS), Lennox‐Gastaut syndrome (LGS), epileptic encephalopathy with continuous spike‐and‐wave during sleep (CSWS), and early‐onset epileptic encephalopathies (EOEEs).^6^ Additionally, the plasticity of brain function should also be considered. Therefore, clinical characteristics, such as semiology of seizures and epileptic encephalopathy, as well as psychomotor developmental and surgical outcome in children with early‐onset seizures would differ from older children or adults with FCD type II.^7^, ^8^ In this study, we analyzed the pre‐ and postsurgery data from 112 young children (epilepsy onset ≤6 years of age) with FCD type II to investigate the clinical features, as well as their seizure and developmental outcomes after surgery.

## 2 METHODS

### 2.1 Inclusion criteria of patients

We analyzed data from patients with refractory epilepsy who underwent surgical treatment from May 2014 to April 2018 in the Children Epilepsy Center of Peking University First Hospital. Patients fulfilled all the following criteria were included: (a) age of seizure onset ≤6 years old; (b) drug‐resistant refractory epilepsy according to the criteria defined by the International League Against Epilepsy (ILAE);^9^ (c) focal lesions of FCD type II were revealed in brain magnetic resonance imaging (MRI), such as cortical thickness, signal hyperintensity (mainly on T2FLAIR and T2‐weighted sequences), blurring of gray‐white matter, and “transmantle” sign;^10^ (d) histological confirmation of FCD type II (including FCD IIa and IIb) based on resected specimens; and (e) follow‐up at least 6 months after surgery.

### 2.2 Presurgical information

We reviewed clinical features, including gender, age at seizure onset, epilepsy exposure duration (calculated as the interval from age at seizure onset to age at surgery), seizure frequency, seizure types, number of antiepileptic drugs (AEDs), and history of diagnosis with any of epileptic encephalopathies. The seizure types were classified according to the operational classification by ILAE.^11^ We classified epileptic encephalopathy as IS (with onset age under 1 year, epileptic spasms, psychomotor delay, and hypsarrhythmia patterns on EEG),^5^, ^12^ LGS (with onset age in childhood, multiple seizures types including tonic seizures, atonic seizures or atypical absences, cognitive impairment, as well as generalized slow spike‐wave patterns and bilateral fast rhythm patterns during slow sleep on EEG),^12^ CSWS (with onset age in childhood, neurocognitive regression, and at least 85% continuous spikes‐and‐waves activities of slow‐wave sleep on EEG),^12^ or EOEEs (with onset from early infancy, recurrent seizures, and developmental delay, including Ohtahara syndrome, early myoclonic epileptic encephalopathy, malignant migrating partial seizures in infancy, and others).^13^

The presurgical evaluation included brain MRI, FDG‐PET (fluorodeoxyglucose positron emission tomography), PET‐CT/MR image coregistration, long‐term video EEG recordings of habitual seizures, and neuropsychological assessments. All patients underwent 3.0T MRI of epileptic sequences, including T1‐weighted, T2‐weighted, FLAIR, and DWI sequencing in the axial, sagittal, and coronal planes. Interictal/ictal scalp EEGs were recorded, and the electrodes were arranged according to the international 10‐20 systems. EEG data were categorized as ipsilateral (to the FCD lesion) or as bilateral/generalized. Ages and Stages Questionnaire version 3 (ASQ‐3)^14^ and the Griffiths Mental Development Scales were used to assess each patient's developmental level. Patients were divided into the following four categories: normal (DQ > 85), mild delay (70 < DQ ≤ 85), moderate delay (35 < DQ ≤ 70), and severe delay (DQ ≤ 35). Participants were dichotomized based on the results: normal/mild delay (DQ > 70) or moderate/severe delay (DQ ≤ 70).

### 2.3 Surgery

We collected the following surgical related data: intracranial EEG recordings (if required), age at surgery, operative site and procedures, and histopathologic diagnoses. The surgical resections were classified as follows: unilobar resection, multilobar resection, and hemispherotomy.

### 2.4 Postsurgical data

The seizure outcome was classified using the Engel score^15^ according to the medical records from the last follow‐up. The postoperative seizure outcome was classified according to Engel: I, free of seizures; II, occasional seizures <2 seizures/year or >90% seizure reduction; III, 90%‐75% reduction in seizure frequency; and IV, <75% reduction in seizure frequency.

We evaluated the developmental outcome after surgery using ASQ‐3 and the Griffiths Mental Development Scales. Based on rank changes in DQ scores, the outcomes were divided into three subcategories, that is, improved, declined, or stabilized.^16^ If the rank was ascending according to the scores, we judged to be improved, otherwise, to be declined or stabilized.

### 2.5 Ethics and informed consent

This study was approved by the institutional review board of the ethics committee of Peking University First Hospital. The parents of all participants had been provided written informed consent for the use of the children's information for scientific purposes.

### 2.6 Statistical analysis

Categorical variables were summarized as numbers and as percentages of the total number of patients in each category, including the history of epileptic encephalopathy, seizure types (with focal seizures only or not), lateral consistency of interictal/ictal EEG discharges with lesions, development before surgery (DQ > 70 or DQ ≤ 70), developmental outcomes, seizure outcomes, extent of lesions, and histopathology. Continuous variables including age at epilepsy onset and surgery, epileptic duration, number of AEDs, and seizure frequency. Since these were all non‐normally distributed variables, they were expressed as medians and ranges. Factors were collected to analyze the correlation with epileptic encephalopathy including age at onset and surgery, seizure frequency, exposure duration, lateral consistency of interictal/ictal EEG discharges, the extent of lesions, and histopathology. For neurodevelopmental levels and outcomes, we also analyzed the differences in number of AEDs, seizure types, and history of epileptic encephalopathy. In addition to the above variables, the developmental level before surgery was considered relevant factors in the analysis of seizure outcomes. The chi‐square test and Fisher exact test were used to compare categorical data. The Mann‐Whitney *U* test was used to compare continuous variables between two groups. Variables with a significance level <0.1 in the initial univariate analysis were then tested in multivariate logistic regression analysis. Kaplan‐Meier survival analysis was performed to calculate differences in the seizure‐free rate after surgery between two groups. Spearman's correlation analysis was used for continuous data. All statistical analyses were performed using SPSS 24.0 for Windows (SPSS Inc).

## 3 RESULTS

### 3.1 Clinical characteristics of young children with FCD type II

The study included 112 children, 76 boys and 36 girls, who met the inclusion criteria. The demographic and clinical characteristics of the patients were summarized in Table 1. The median age at seizure onset was 0.9 years (3 days‐5.9 years). In 51.8% (58/112) of patients, seizure began before 1 year of age, 1‐3 years of age in 27.7% (31/112), and 20.5% (23/112) after 3 years. The median age at surgery was 4.1 years (9.2 months‐16.2 years), and the median epilepsy exposure duration before surgery was 2.3 years (3.6 months‐14.3 years). Patients usually had frequent seizures, with a median of five seizures per day (once/week to more than fifty/day) despite receiving multiple AEDs (median number = 5, 2‐12).

**Table 1:** Clinical features related to history of epileptic encephalopathy, developmental level, and surgery outcomes (Data were showed n (%) or median (range))

	Total (n = 112)	History of EE		Seizure outcomes		Development before surgery		Developmental outcomes	
		Without (n = 98)	With (n = 14)	Seizure‐free (n = 99)	Seizure ongoing (n = 13)	Normal/mild delay (n = 57)	Moderate/severe delay (n = 55)	Improvement (n = 4)	Stable (n = 44)
Age at onset (y)	0.9 (0.01‐5.9)	1.2 (0.01‐5.9)	0.4 (0.03‐1.5)^a^	0.9 (0.01‐5.9)	0.9 (0.1‐5.8)	1.7 (0.01‐5.8)	0.6 (0.01‐5.9)^f^	1.6 (0.44‐2.8)	0.5 (0.01‐5.9)
Seizure frequency (n/day)	5 (0.1‐50)	5.3 (0.1‐50)	3.3 (0.3‐25)	4.5 (0.1‐35)	7 (1‐50)	4.5 (0.1‐25)	6.0(0.1‐50)	2.9 (0.2‐23)	7.3 (0.3‐50)
Age at surgery (y)	4.1 (0.8‐16.2)	4.2 (0.8‐16.2)	2.0 (1.2‐6.6)^b^	4.2 (0.8‐16.2)	3.3 (1.2‐14.1)	4.5 (0.8‐14.2)	3.9 (0.8‐16.2)	4.7 (4.1‐6.8)	2.8 (0.8‐7.3)
Exposure duration (y)	2.3 (0.3‐14.3)	2.5 (0.3‐14.3)	1.7 (0.5‐5.2)	2.5 (0.3‐14.3)	2.2 (1.0‐9.4)	2.3 (0.3‐12.2)	2.3 (0.4‐14.3)	2.8 (2.0‐6.2)	2.1 (0.4‐6.3)
Numbers of AEDs	5 (2‐12)	‐	‐	‐	‐	4 (2‐8)	5 (2‐12)^g^	‐	‐
Development before surgery									
Normal/mild delay	57 (50.9)	55 (56.1)	2 (14.3)^c^	54 (54.5)	3 (23.1)	‐	‐	‐	‐
Moderate/severe delay	55 (49.1)	43 (43.9)	12 (85.7)	45(45.5)	10 (76.9)^e^	‐	‐	‐	‐
History of EE									
Without	98 (87.5)	‐	‐	88 (88.9)	10 (76.9)	55 (96.5)	43 (78.2)	4 (100)	32 (72.7%)
With	14 (12.5)	‐	‐	11 (11.1)	3 (23.1)	2 (3.5)	12 (21.8)^h^	0	12 (27.3)
Seizure types									
Focal seizures only	78 (69.6)	‐	‐	70 (70.7)	8 (61.5)	45 (78.9)	33 (60)		
Other seizures	34 (30.4)	‐	‐	29 (29.3)	3 (38.5)	12 (21.1)	22 (40)^i^		
Interictal EEG discharges									
Ipsilateral	98 (87.5)	86 (87.8)	12 (85.7)	87 (87.9)	11 (84.6)	51 (89.5)	47 (85.5)		
Bilateral	14 (12.5)	12 (12.2)	2 (14.3)	12 (12.1)	2 (15.4)	6 (10.5)	8 (14.5)		
Ictal EEG									
Ipsilateral	101 (90.2)	93 (94.9)	8 (57.1)	88 (88.9)	13 (100)	53 (93)	48 (87.3)		
Bilateral	11 (9.8)	5 (5.1)	6 (42.9)^d^	11 (11.1)	0	4 (7)	7 (12.7)		
The extent of lesions									
Single lobes	84 (75)	76 (77.6)	8 (57.1)	75 (75.8)	9 (69.2)	42 (73.7)	42 (76.4)	4 (100)	31 (70.5)
Multiple lobes	28 (25)	22 (22.4)	6 (42.9)	24 (24.2)	4 (30.8)	15 (26.3)	13 (23.6)	0	13 (29.5)
Histopathology									
FCD IIa	36 (32.1)	29 (29.6)	7 (50.0)	30 (30.3)	6 (46.2)	17 (29.8)	19 (34.5)	1 (25)	16 (36.4)
FCD IIb	76(67.9)	69 (70.4)	7 (50.0)	69 (69.7)	7 (53.8)	40 (70.2)	36 (65.5)	3 (75)	28 (63.6)

Statistical analysis included Mann‐Whitney test, Kaplan‐Meier survival analysis, and Pearson chi‐square test with Fisher's exact test, as appropriate.

Abbreviation: y, years; AED, antiepileptic drug; EE, epileptic encephalopathy; EEG, electroencephalograph; MRI, Magnetic resonance imaging; FCD, focal cortical dysplasia

(a‐d) Comparison between without/with epileptic encephalopathy, if *P* < .05: ^a^ *P* = .001, ^b^ *P* = .001, ^c^ *P* = .003, ^d^ *P* = .001.

(e) Comparison between seizure‐free group and seizure ongoing group, if *P* < .05: ^e^ *P* = .032.

(f‐i): Comparison between the two preoperatively developmental level group, if *P* < .05: ^f^ *P* < .001, ^g^ *P* < .001, ^h^ *P* = .003, ^i^ *P* = .029.

As for seizure types during disease course, 69.6% (78/112) of patients had only focal seizures, while 11 (9.8%) had both focal seizures and generalized seizures, including myoclonic seizures, tonic seizures, atypical absence seizures, and generalized tonic‐clonic seizures (GTCS). We found that 20.5% (23/112) of patients had epileptic spasms, and five of whom had only epileptic spasms without any other seizure types.

**Figure 1:**
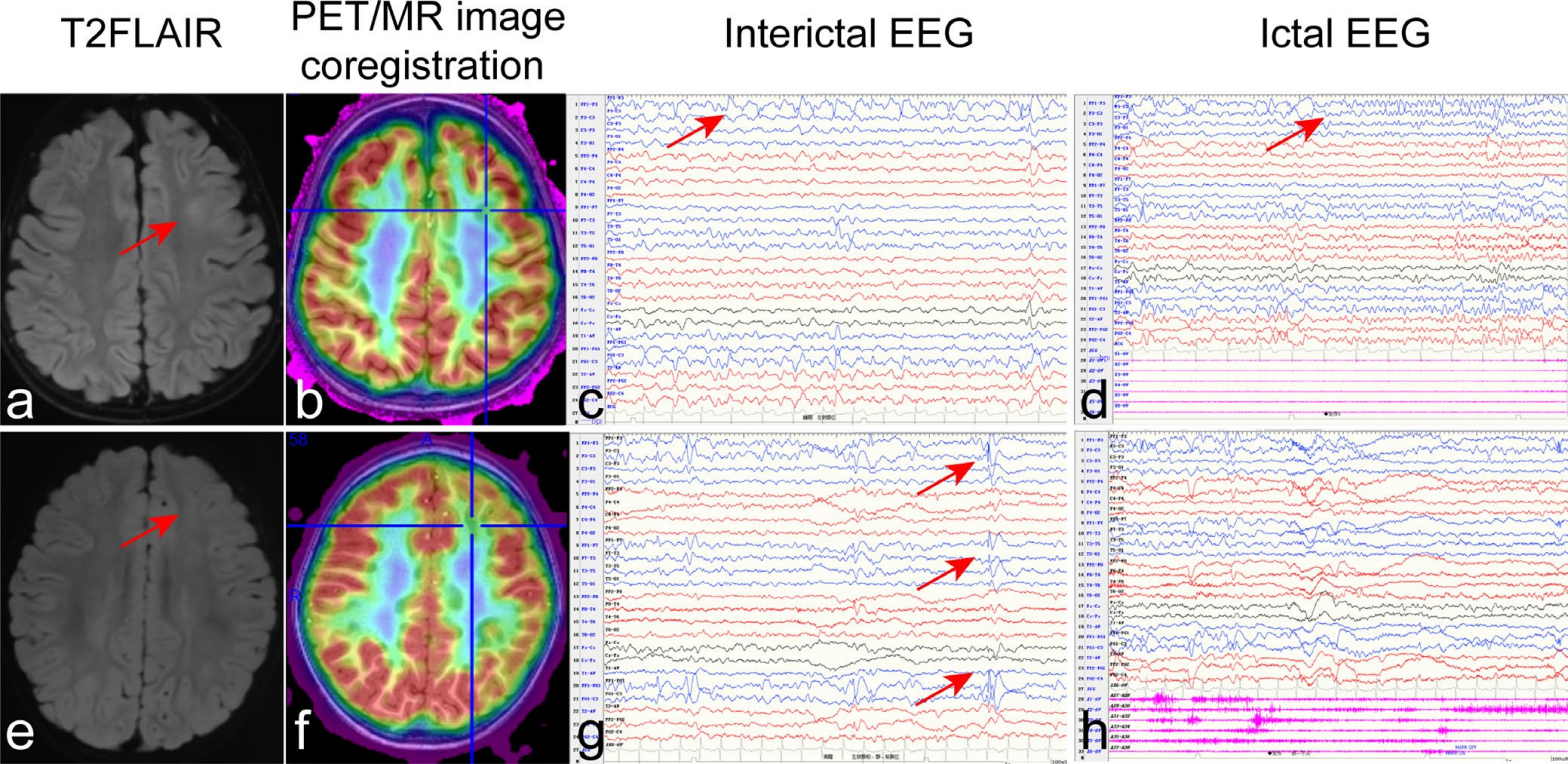
Neuroimages and EEGs in two patients. a‐d, FCD IIb in a boy with onset age of 1.8 y. (a) Focal signal hyperintensity and blurring of gray‐white matter in left frontal area was shown on T2FLAIR image (red arrow). (b) The region with lower metabolism was presented on coregistration image. (c) The interictal EEG showed sharp waves and sharp slow waves in left frontal areas (red arrow). (d) Focal seizures with left frontal onset on ictal EEG were monitored (red arrow). e‐h, FCD IIa in a boy with onset age of 7 months. In frontal lobe, burring of gray‐white matter on T2FLAIR image (e, red arrow) and lower metabolism on coregistration image (f) were showed. (g) The interictal EEG showed spikes, sharp waves and sharp slow waves in left frontal and temporal areas (red arrow). (h) Generalized spasm was monitored on ictal EEG. (The blue waveforms represented the left side, and the red represented the right side.)

According to the scalp EEG monitoring during presurgical evaluation, most of the patients (87.5%) showed interictal epileptic discharges ipsilateral or predominantly lateral to the lesion, whereas fourteen cases (12.5%) showed bilateral interictal discharges. A total of 701 seizures were recorded during the EEG monitoring in 112 patients (1‐30 seizures per case, median = 4). Among these seizures, 92.5% were recognized as focal seizures with onset ipsilateral to the lesion, which included focal epileptic spasms, focal myoclonic, hyperkinetic, automatisms, focal tonic, and focal clonic seizures. And 7.5% (53/701) were bilateral onsets on ictal EEG, including epileptic spasms (84.9%, 45/53) and myoclonic seizures (15.1%, 8/53) (Figure 1).

Unilobar resection was performed in 84 patients (75%), with resection of frontal lobe in 56 (66.7%, 56/84), parietal lobe in 18 (21.4%, 18/84), temporal lobe in 8 (9.5%, 8/84), and occipital lobe in 2 (2.4%, 2/84). Multilobar resection was performed in 27 patients (24.1%) and hemispherotomy in 1 patient (0.9%). Histopathology of brain tissue was confirmed to be FCD IIa in 36 cases (32.1%) and FCD IIb in 76 cases (67.9%).

### 3.2 Epileptic encephalopathies and related factors

IS was diagnosed in 14 (12.5%) children before the surgery. None of the children in our cohort were diagnosed with LGS, CSWS, or EOEE. Both the age of onset and age of surgery were younger in children with IS than children without epileptic encephalopathy (0.4 years vs. 1.2 years, *P* =* *.001; 2.0 years vs. 4.2 years, *P* = .001). The proportion of patients with bilateral discharges on ictal EEG was much higher in children with IS (42.9% vs 5.1%, *P* =* *.000). There was no difference in exposure duration (*P* = .075), seizure frequency (*P* = .352), lateral consistency of interictal EEG discharges (*P* = .687), extent of the FCD II lesions (*P* = .110), or histopathology (*P* = .138) between patients with and without epileptic encephalopathies (Table 1). Multifactorial regression analysis showed that younger age of seizure onset (OR = 0.213, 95%CI: 0.048‐0.942, *P* = .041) and bilateral ictal EEG discharges (OR = 9.059, 95%CI: 2.017‐40.684, *P* = .004) were associated with epileptic encephalopathy.

### 3.3 Seizure outcomes after surgery

Patients were followed 6 months to 4.5 years (median 1.0 years) after surgery. At the last follow‐up, 88.4% (99/112) of patients were seizure‐free, while the remaining including four patients with Engel II, 2 with Engel III, and 7 with Engel IV seizures. Of 13 patients with ongoing seizures, eight had seizures shortly after surgery, three had recurrence within 6 months after surgery, and two had recurrence 6 months‐1 year later. And of 14 cases with epileptic encephalopathy, 78.6% (11/14) achieved seizure‐free and 3 with Engel IV.

Comparing the seizure group versus seizure‐free group, we found a higher percentage of developmental delays in seizure group before surgery (76.9% vs 45.5%; *P* = .032). There is no significant difference between the two groups in any of the following factors: age at onset (*P* = .989), age at surgery (*P* = .510), epilepsy exposure duration (*P* = .605), seizure frequency (*P* = .237), seizure types (*P* = .554), history of epileptic encephalopathy (*P* = .214), lateral consistency of EEG (Interictal V‐EEG: *P* = .694, ictal V‐EEG: *P* = .219), extent of lesion (*P* = .643), and histopathology (*P* = .218) (Table 1).

### 3.4 Psychomotor development before and after surgery

Before the surgery, fifty‐seven (50.9%) patients showed normal development milestones or mild delay, while 55 (49.1%) showed moderate or severe delay. Univariate analysis showed that patients with moderate or severe delay had younger age of epilepsy onset (0.6 years vs. 1.7 years, *P* = .000), had tried more AEDs (5 vs 4, *P* = .000), had higher rates of epileptic encephalopathy (12% vs 2%, *P* = .003), and had more seizure types (22% vs 12%, *P* = .029). We did not find differences in seizure frequency (*P* = .208), age at surgery (*P* = .127), epilepsy exposure duration (*P* = .596), lateral consistency of EEG (Interictal V‐EEG: *P* = .520, ictal V‐EEG: *P* = .310), extent of lesion (*P* = .743), and histopathology (*P* = .593) (Tables 1). Correlation analysis showed that the DQ level was significantly negatively correlated with prolonged epileptic duration (*r* = −.400, *P* = .002). Multivariate analysis showed that earlier onset (OR = 0.740, 95%CI: 0.572‐0.959, *P* = .023) and a history of epileptic encephalopathy (OR = 5.160, 95%CI: 1.058‐25.152, *P* = .042) were more likely to predict developmental delay.

Then, we focused on the developmental outcome of the 55 patients who showed moderate and severe developmental delays before surgery. There were 48 patients with complete paired assessment data (both before and after surgery) available. Analysis showed that 91.7% (44/48) was stable after surgery, while four cases (8.3%, 4/48) showed improvement. In this cohort, all four patients with improvement were preoperatively moderate delay, had no history of epileptic encephalopathy, and achieved seizure‐free after surgery, and the lesions were limited in single lobes. And they tended to have older age of epilepsy onset and surgery, and lower seizure frequency before surgery. Patients with epileptic encephalopathy all showed stable level of development after surgery. For those with stable development, although they had acquired new skills according to ASQ‐3, the DQ ranking did not show improvement at 6 months after surgery. And 77.3% (34/44) of them achieved seizure‐free. (Table 1).

## 4 DISCUSSION

The structure and function of brain maturate continuously throughout early life shaped by the interaction of genetics, environment, and experiences.^17^ For young children, the immature brain, with higher expression of excitatory neurotransmitters, remodeling synaptic patterns, incomplete myelination, and deficient local and longer‐range connections, makes it vulnerable to seizure and increasing synchronization.^18^ And the lesion of FCD II might result from aberrant proliferation or apoptosis of cells during the period of gestation.^19^, ^20^ The mechanism of generation is related to brain somatic variations.^21^, ^22^ The lesion could have adverse impact on the developing brain. Especially for patients with earlier seizure onset, the influence of lesion on the brain function might be much serious.

### 4.1 Clinical characteristics of younger children with FCD type II different from those of older children or adults

In term of seizure types, epileptic spasms were common in young children and 61.6% of spasms showed bilateral onset on ictal EEG in our cohort. With increasing age, the proportion of spasms was reduced.^23^, ^24^, ^25^ In the cohort reported of 62 patients with FCD II with an average onset age of 7 years, there was no patient having epileptic spasm.^23^ The epileptic spasm may originate from the local cortex and rapidly spread through the subcortical structure to the bilateral hemispheres, resulting in the clinical seizures. It is age‐related and may be associated with immature brain function in young children.^26^ In term of scalp EEG, FCD II usually exhibits some characteristic EEG activities. The interictal EEG frequently showed rhythmic discharges concordant with the anatomic lesions. And the dischargers sometimes spread to adjacent areas, especially in type IIb.^23^, ^27^ The ictal EEG patterns reveal poly‐spikes and low‐voltage fast activities. Delta brushes could be occasionally represented at seizure onset,^27^, ^28^, ^29^ whereas the EEG abnormalities tend to be more diffuse and nonlocalized in young children.^30^ We found that 12.5% cases showed bilateral discharges on interictal EEG and 11% with bilateral onset on ictal EEG. The diffuse EEG discharges in young children may be due to lower development level of young children in which local lesions more likely cause synchronization or comprehensive discharges.^31^ While, for adult with FCD, EEG discharges tend to be more regional and 95% cases present concordance of ictal with interictal EEG abnormalities.^32^

### 4.2 Epileptic encephalopathy not uncommon in young children with FCD type II

Among the children in this cohort, 12.5% cases had a history of epileptic encephalopathy, all of which were IS. Krsek et al^15^ reported that in 16 FCD II children with onset age under 7 years, two (12.5%) had a history of IS. Kwon et al^16^ found 32% FCD cases (30.2% FCD II) with onset under 5 years presented as epileptic encephalopathy, including eight IS and sixteen LGS cases. Since the occurrence peak of LGS was 3‐5 years age,^33^ the lower proportion of LGS in our study might result from the earlier surgery for those with IS (the median operation age was 2.0 years). It was hypothesized that the cerebral dysfunction of IS results from the impaired and unbalanced maturational process and the causative factors include abnormal neurogenesis, apoptosis, synaptic pattern, and so on,^34^ which are also related to the generation of FCD lesion. And chronic epilepsy could cause dysfunction of whole‐brain network.^35^, ^36^ Additionally, multivariate analysis in our study showed that earlier onset and bilateral onset on ictal EEG were more frequent in patients with history of IS. Although this made it challenging to identify epileptogenic zones, 76.9% of the patients with IS achieved postoperative seizure‐free, similar to the percentage without IS (88.9%) in our cohort.

### 4.3 Seizure outcome in young children was as favorable as older children or adults

In this study, 88.4% cases achieved seizure freedom at the last follow‐up, similar to that of older children or adults.^37^ The seizures relapse mostly within 12 months after surgery,^38^, ^39^, ^40^ and 48.2% cases in our cohort were followed for less than 1 years, so it still needs longer follow‐up. We observed higher rate of moderate/severe delay before surgery in group of patients not seizure‐free. Low level of development may reflect widespread disturbance of cerebral function.^41^ And seizure outcomes also related to higher rate of history of epileptic encephalopathy and multilobar lesions in our study. Malmgren et al^41^ found that for patients with IQ < 70 before epilepsy surgery, the rate of seizure‐free was lower (about 33.3%), and preoperative IQ level was an independent predictor of seizure outcome at two years follow‐up. In addition, seizure outcomes were reported to relate to other factors. A study of 100 patients with FCD II, onset range from 0 to 30 years of age, suggested that seizure freedom after 81 months follow‐up was associated with FCD type IIb and single lobar lesions.^29^ Wagner et al^38^ found that incomplete resection was a negative predictor to failure of surgery. While, incomplete resection always resulted from colocalization of lesion to functional area.^27^, ^42^ Besides, research from Jin et al^40^ suggested that discharges of EEG 3‐6 months after surgery and habitual acute postoperative seizure (APOS) could predict seizure recurrence. Fauser et al^39^ found that younger age at surgery and shorter epilepsy duration were associated with higher seizure‐free percentages in 211 patients with FCD (onset age ranges from 0 to 60 years old, including 40% FCD II cases and following up at least two years). However, it was still controversy. One study on FCD II patients under 6 years of onset age, comparing two groups underwent surgery at <6 and >20 years old, the results showed no significant difference in seizure outcome between the two groups.^43^

### 4.4 Moderate to severe psychomotor delay common was common, with unsatisfactory developmental outcome at 6 months after surgery

Nearly half (49.1%) cases were moderate to severe development delay before surgery, which was higher than that of older children (<16%) in our cohort.^23^, ^43^ We found the delay was associated with earlier onset, more AEDs, history of epileptic encephalopathies, multiple seizure types, and longer epileptic duration. Kimura et al^44^ analyzed risk factors of cognitive impairment in 77 FCD patients with childhood‐onset epilepsy (including 68 FCD II), the results showed that frequent seizures, epileptic spasms, and earlier onset epilepsy were risk factors. Moreover, developmental delay might also relate to larger dysplastic lesions in children.^23^, ^45^

Whether the children will show developmental improvement after surgery is an important factor to consider in the presurgery counseling. In our study, for young children with preoperatively moderate to severe developmental delay, the developmental outcome at 6 months after surgery was not satisfactory. Only 8.3% of cases achieved improvement 6 months after surgery. Although all children did acquire new skills according to ASQ‐3. It should be noted that DQ reflected the developmental level relative to normal age‐matched children and no improvement in DQ rank suggest any change in development.^46^ Therefore, the percentage of improvement after surgery may be underestimated, and the development outcome still required longer follow‐up and evaluation. We found all children with developmental improvement had no history of epileptic encephalopathy, were moderate (not severe) delay before surgery, and had single lobar lesions as well. A study on the cognitive outcome of epileptic surgery in children with follow‐up 0.2 to 17.4 years showed that the development level mostly remained stable and only 20%‐30% of children had cognitive improvement after surgery,^47^ and only patients with seizure‐free showed improvement.^16^, ^48^ It might due to the reduced effects of frequent seizures, AEDs, and devastating EEG discharges on brain function. And the removal of epileptogenic zone could also interrupt its reaction to the surrounding region, which could carry chance to achieve neurodevelopmental progress.^46^ Especially for young children in rapid developmental stage, earlier acquisition of seizure‐free is necessary for neurodevelopment, and long‐term outcome might be better with strong brain plasticity. Chen et al^49^ conducted average 21.5 months of follow‐up study in 30 FCD patients and found that earlier surgery (epilepsy duration <2 years) and complete resection were beneficial to the improvement of development and quality of life.

This study had some limitations. It may be biased as it was a retrospective study that was conducted at a single tertiary epilepsy treatment center and with relatively small number of patients. In term of neurodevelopment, it would be more comprehensive to interpreting the developmental outcomes by analyzing changes in different aspects of psychomotor development and the quality of life. And more valid and applicable assessment for different age of patients might be useful for longitudinal follow‐up. Importantly, longer follow‐up after surgery is needed to better assess outcomes.

## 5 CONCLUSIONS

For young children with FCD type II, epileptic spasms and bilateral EEG discharges were more common than older children or adults. Young children also tend to present with epileptic encephalopathies and show moderate to severe developmental delay before surgery. The seizure outcome was favorable after surgery, even for those with epileptic encephalopathy and severe developmental delay, but of those, developmental outcome at 6 months after surgery was not satisfactory.

## CONFLICT OF INTEREST

None of the authors have any conflict of interest to disclose.
